# Effects of Peppermint Essential Oil on Learning and Memory Ability in APP/PS1 Transgenic Mice

**DOI:** 10.3390/molecules27072051

**Published:** 2022-03-22

**Authors:** Xiaofan Lv, Yueyang Feng, Rui Ma, Yin Tang, Ye Li, Donghong Cui, Yani Wu

**Affiliations:** 1 School of Design, Shanghai Jiao Tong University, Shanghai 201100, China; fanya.l@sjtu.edu.cn (X.L.); yueyangf@sjtu.edu.cn (Y.F.); mrm9526@sjtu.edu.cn (R.M.); tangyin01@sjtu.edu.cn (Y.T.); mm90921@163.com (Y.L.); 2 Mental Health Centre, Medical College, Shanghai Jiaotong University, Shanghai 200240, China; donghong.cui@gmail.com

**Keywords:** peppermint essential oil, learning and memory, metabonomics, hippocampal

## Abstract

Objective: To explore the effect and mechanism of peppermint essential oil on learning and memory ability of APP/PS1 transgenic mice. Methods: Morris water maze test and shuttle box test were used to explore the changes in learning and memory ability of APP/PS1 transgenic mice after sniffing essential oil. The cellular status of neurons in the hippocampal CA1 region of the right hemisphere, Aβ deposition, oxidative stress level, and serum metabonomics were detected to explore its mechanism. Results: Sniffing peppermint essential oil can improve the learning and memory ability of APP/PS1 transgenic mice. Compared with the model group, the state of neurons in the hippocampal CA1 region of the peppermint essential oil group returned to normal, and the deposition of Aβ decreased. The MDA of brain tissue decreased significantly, and the activity of SOD and GSH-PX increased significantly to the normal level. According to the results of metabonomics, it is speculated that peppermint essential oil may improve cognitive function in AD by regulating arginine and proline metabolism, inositol phosphate metabolism, and cysteine and methionine metabolism.

## 1. Introduction

Alzheimer’s disease is a common neurodegenerative disease in the elderly, which is irreversible and fatal [1]. Common symptoms of Alzheimer’s disease include cognitive impairment and decline in learning and memory ability [2]. Every 3 s, there will be a new patient with Alzheimer’s disease in the world, and the number of patients with Alzheimer’s disease will increase with the ageing of the population. It is estimated that by 2050 there will be more than 100 million AD patients in the world [3]. However, at present, there is no systematic treatment for Alzheimer’s disease, and the existing therapeutic drugs such as donepezil and memantine have the disadvantages of high price, poor efficacy, and many side effects [4]. Therefore, the early prevention of Alzheimer’s disease is particularly important [5]. In the prevention and treatment of Alzheimer’s disease, some natural therapies using pure plant extracts and active ingredients have been paid more and more attention [6,7], such as the application of aromatherapy—for example, sniffing the aroma of green tea [8], using aromatic essential oils such as bath salts, and so on [9,10].

Peppermint essential oil is a secondary metabolite extracted from *Mentha × piperita* L. It is a colourless or yellowish oily liquid. Its main volatile components are menthol, menthone, 1–8 cineole, etc. [11]. Mint essential oil has a strong refreshing effect, and traditional Chinese medicine believes that it has the effect of “pungent to diverge, cool to clear heat; clear the leader” [12]. Evidence suggests that peppermint aroma can improve learning and memory impairments. It has been proved that mint compound essential oil sniffing therapy can improve the cognitive function of patients with mild cognitive impairment by reducing the content of serum AchE [13]. AchE is a hydrolase of acetylcholine and an important marker enzyme reflecting the function of the brain cholinergic system. The decline in learning and memory ability is directly related to the decrease in the activity of acetylcholinergic neurons.

Therefore, this study investigated the changes in spatial memory, learning and memory ability, amyloid-β (Aβ) deposition around neurons in the hippocampal CA1 region of the right hemisphere, and oxidation indexes in vivo in APP/PS1 transgenic mice (AD model mice) after sniffing peppermint essential oil. Metabonomics was used to identify the differential metabolites in serum of mice to explore the effect of peppermint essential oil on learning and memory ability of APP/PS1 transgenic mice by sniffing, so as to provide a reference value for further study of aromatherapy for improving and preventing Alzheimer’s disease.

## 2. Materials and Methods

### 2.1. Materials

#### 2.1.1. Reagents and Equipment

Main reagents: Peppermint essential oil and rosemary essential oil were provided by the aromatherapy laboratory of Shanghai Jiaotong University (Shanghai, China). DAB concentrated kit and broad-spectrum secondary antibody were purchased from Shanghai Changdao Biotechnology Co., Ltd. (Shanghai, China). Total protein (BCA) test box, MDA, SOD, and GSH-PX test box were purchased from Nanjing Jiancheng Biological Engineering Institute (Nanjing, China). Methanol, methoxyamine hydrochloride, chloroform, pyridine, L-2-chlorophenyl alanine, and n,o-bis-trimethylsilyl-trifluoroacetamide (BSTFA) that contained 1% trimethylchlorosilane (TMCS) were provided by Analysis and Testing Centre chromatograph and Pegasus 4D TOF-MS.

Main equipment: Laboratory Equipment Corporation; 7890B-5977B GC-MS, Agilent (Shanghai, China). TGL-16M high-speed freezing centrifuge, Hunan Xiangyi Laboratory Instrument Company Limited (Hunan, China). LNG-T88 vacuum centrifugal concentrator, Taicang Huamei Biochemical Instrument Factory (Jiangsu, China). ECLIPSENi positive microscope, DS-Ri2 microscopic image analysis system, NIKON Instruments (Shanghai, China).

#### 2.1.2. Animals and Groups

Learning and memory impairment model mice induced by APP/PS1 transgenic (model mice) and normal C57BL/6J mice (normal mice) of the same genetic background, 6-month-old, license number SCXK (Su) 2016-0010, were provided by Changzhou Cavens Laboratory Animal Co., Ltd. (Jiangsu, China) The normal group used normal mice; the model group, peppermint essential oil group, and positive group used model mice, with 8 mice in each group. The mice of the peppermint essential oil group sniffed peppermint essential oil, while the mice of the positive group sniffed rosemary essential oil (efficacy was determined by animal and volunteer experiments [14]), 2 times/day, 1 h/time, for 21 days.

### 2.2. Methods

#### 2.2.1. Morris Water Maze

All the animal experiments were conducted in the Mental Health Centre affiliated with the Medical College of Shanghai Jiaotong University. On the fifth day of sniffing, a total of 32 mice were tested by Morris water maze; the positioning cruise test was conducted in the first five days, and the space exploration test was conducted on the sixth day.

The platform (diameter, 80 mm) was placed in the second quadrant of the water maze (height × diameter, 1100 mm × 1500 mm) slightly below the horizontal plane, and the position was kept unchanged. In the positioning cruise test, the mice were put into the water facing the pool wall at a fixed entry point. Within 120 s, the mice that reached the target platform were made to stay on the platform for 30 s, and the mice that failed to reach the platform were placed on the platform to remember for 30 s. The time for mice to find the platform was recorded as the escape latency, and the movement trajectory of mice was recorded. On day 6, the underwater platform was removed for the space exploration test. The times of each mouse crossing the original platform position and the residence time in the original platform quadrant within 120 s were recorded, and the movement track of each mouse was also captured.

#### 2.2.2. Shuttle Box Test

This test used the shuttle box (length × width × height, 450 mm × 250 mm × 260 mm), and was divided into two days. The mice were trained on the first day, and the trained mice were tested on the second day. Each mouse was placed into the shuttle box on one side and was given a sound and light warning for 5 s. When the mouse escaped to the other side through the middle hole within that time, it was regarded as an active escape. If the mouse remained in this area, the bottom iron net (0.3 mA) was added 5 s later, so that the mouse had painful current stimulation (while the sound and light warning continued) until the mouse escaped to the other side of the box (no electricity), it was regarded as a passive escape. Each mouse performed 20 escape tests, and the number of active escapes and the latency of active escape were recorded. After the experiment of each mouse, the excretion collection plate was cleaned to prevent the remaining odour from stimulating the mice in the follow-up experiment.

#### 2.2.3. Sample Collection and Pretreatment

After the behavioural experiments, the mice were anaesthetized with ether to take eyeball blood, and the serum was separated (4 °C, 3000 r/min, 15 min) and stored at −80 °C for metabonomic testing. When in use, the serum was thawed at room temperature. An amount of 100 µL serum was taken to a centrifuge tube, 300 µL methanol-chloroform (3:1, *v/v*) and 10 µL L-2-chlorophenyl alanine (0.3 mg/mL) were added, and the centrifuge tube was shaken for 30 s, and the serum was placed at 20 °C for 20 min. The sample was centrifuged for 10 min (4 °C, 12,000 r/min), 300 µL supernatant was taken into a 1.5 mL autosampler vial, and the sample was concentrate in a vacuum and dried with nitrogen. An amount of 80 µL pyridine solution of methoxyamine hydrochloride (15 mg/mL) was added, and the vial was sealed and placed on a shaking bed for one night at room temperature. Then, the vial was opened, 80 µL BSTFA (including 1% TMCS) was added, and the vial was sealed and placed in a thermostatic box for 60 min at 70 °C. The sample was used for GC-MS detection. Part of the fresh brain tissue of the right hemisphere of the mice was fixed with 10% formalin for 24 h and made into HE staining pathological sections. The other part was used for kit detection.

#### 2.2.4. Parameters of GC-TOFMS

1. Essential oil composition determination.

DB-5MS capillary column was used (length 30 m, diameter 250 µm, thickness 0.25 µm). The starting temperature of the column was set to 60 °C for 4 min and increased to 240 °C at 8 °C/min for 5 min. The carrier gas was helium with a flow rate of 1 mL/min. Interface and ion source temperatures were 250 °C and 230 °C, respectively. The ionization mode of the mass spectrometer was EI, and the ionization voltage was 70 eV. Mass scan range was 15~550 amu.

2. Serum samples.

The starting temperature of the column was set to 80 °C for 2 min, increased to 180 °C at 10 °C/min, increased to 240 °C at 5 °C/min, and increased to 290 °C at 25 °C/min for 9 min. Interface and ion source temperatures were 270 °C and 220 °C, respectively. In full scan mode, the scan range was 30–550 *m*/*z*. and the serum sample solvent was delayed by 7.5 min.

#### 2.2.5. Data Processing and Analysis

The results of behavioural tests and test indexes were analysed and processed by SPSS 26.0, and the data are expressed as mean ± standard deviation ($\bar{x}$ ± S). The comparison between multiple groups was processed by single-factor analysis of variance, and the comparison between the two samples was analysed by LSD test. Metabonomics data were imported into SIMCA after the original data are processed by LECO chroma TOF software for multidimensional statistical analysis. The specific standards of potential differential metabolites are variable importance in the projection (VIP) > 1 and *p* < 0.05 (according to *t*-test). Substance was identified by the NIST database and the database of Shanghai Jiaotong University Analysis and Testing Centre. The involved metabolic pathways were investigated by the MetaboAnalyst platform.

## 3. Results

### 3.1. Behavioural Experiments

#### 3.1.1. Morris Water Maze

The results of the positioning cruise test (Table 1) showed that the escape latency of mice gradually showed the trend of model group > peppermint essential oil group > positive control group > normal group from the second day. On the third and fourth days, the escape latency of the peppermint essential oil group and the positive group was significantly shortened compared with the model group.

The results of the space exploration test (Table 2) showed that, compared with the normal group, the residence time of mice in the quadrant of the original platform was significantly shortened and that the number of times crossing the original platform was significantly reduced in the model group. Compared with the model group, the residence time in the peppermint essential oil group and the positive group was significantly prolonged and the number of times was significantly increased.

**Figure 1:**
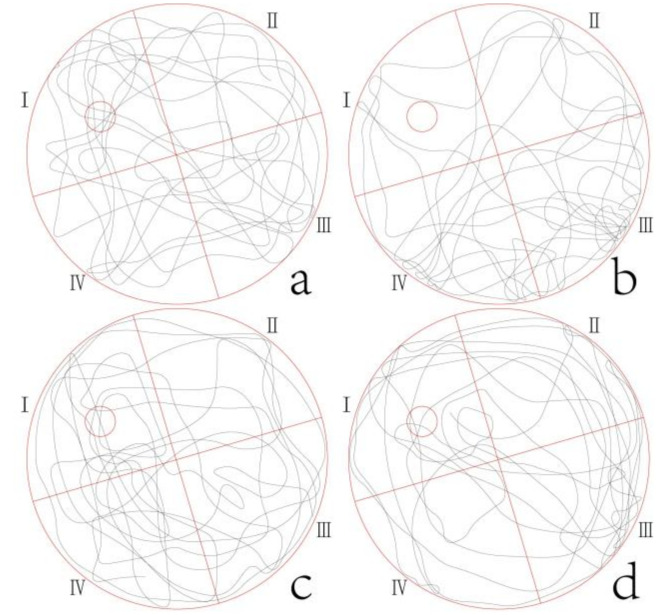
Swimming trajectories of space exploration in normal group (**a**), model group (**b**), positive group (**c**), and peppermint essential oil group (**d**). The quadrant where the original platform was located was set as Ⅰ, and defined quadrants as Ⅱ, Ⅲ and Ⅳ clockwise in turn, with equal area of each quadrant.

The swimming track of mice in each group (Figure 1) showed that the mice in the peppermint essential oil group swam more frequently in Ι quadrant, which was similar to that in the normal group and the positive group. Meanwhile, in the model group, the swimming randomness was stronger, and the swimming track was the least in Ι quadrant.

#### 3.1.2. Shuttle Box Test

The results of the shuttle box test (Table 3) showed that compared with the normal group, the number of active escapes was significantly reduced and the latency of active escape was significantly prolonged in the model group. Compared with the model group, the number was significantly increased and the latency was significantly shortened in the peppermint essential oil group and the positive group.

The results of the Morris water maze and the shuttle box test indicate that sniffing peppermint essential oil can improve the learning and memory ability of mice to a certain extent.

### 3.2. Pathological Features Observation and Detection Results

#### 3.2.1. Pathological Features Observation

**Figure 2:**
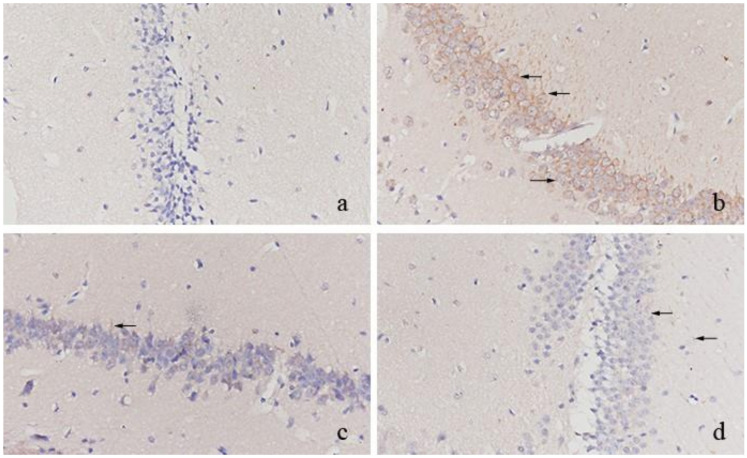
Observation of neurons in brain tissue of mice in normal group (**a**), model group (**b**), positive group (**c**), and peppermint essential oil group (**d**). The arrow points to Aβ precipitation. (HE staining, ×200).

The results of immunohistochemical staining showed that the neurons in the normal group were closely arranged and that no obvious Aβ precipitation was observed (Figure 2a). The neurons were arranged in disorder, and the dark brown Aβ precipitation was observed near the neurons in the model group (Figure 2b). In the sections of the positive group (Figure 2c) and peppermint essential oil group (Figure 2d), the neurons were arranged in order, and Aβ precipitation near mouse brain neurons was significantly reduced; only a small number of Aβ precipitation was observed, and the precipitation colour was relatively light.

#### 3.2.2. Oxidative Stress Index

As can be seen from Table 4, which compares with the normal group, the content of MDA in brain tissues of the model group increased significantly, while the activities of SOD and POD decreased significantly. Compared with the model group, the content of MDA in brain tissues of the peppermint essential oil group and the positive group decreased significantly, while the activities of SOD and POD increased significantly.

### 3.3. Effects of Sniffing Peppermint Essential Oil on Serum Metabolites

#### 3.3.1. PCA and OPLS-DA Analysis

**Figure 3:**
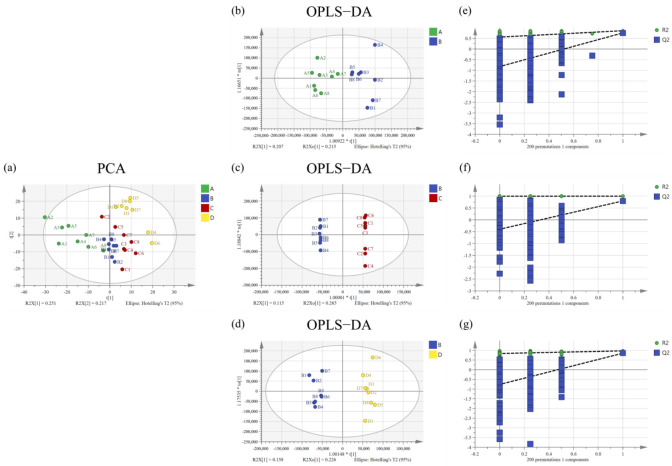
Principal component analysis score plot (PCA), (**a**), orthogonal partial least squared-discriminant analysis (OPLS-DA) score plot (**b**–**d**), and permutation plot (200 permutations), (**e**–**g**) of serum samples collected from the normal group (**A**), model group (**B**), positive group (**C**), and peppermint essential oil group (**D**), (*n* = 8).

The result of principal component analysis (PCA) show that the metabolomic profiles in serum of the normal group, model group, peppermint essential oil group, and positive group were separated (Figure 3). All samples are located in 95% confidence interval.

As the PCA model used the unsupervised mode, the supervised method of orthogonal projections to latent structures discriminant analysis (OPLS-DA) was used to establish the model again, and a permutation test (*n* = 200) was carried out to evaluate whether the model was overfitted. The results are shown in Section 3.4, where metabolites of each of the two groups were found to be separated. Permutation test results indicate that the models were relatively stable and not overfitted.

#### 3.3.2. Potential Biomarkers

The variable importance in projection values (VIP) was calculated from the OPLS-DA, and an independent sample test was used between groups to further identify variables with *p* < 0.05. A total of 36 potential biomarkers were identified in the model group compared with the normal group, in which 5 were up-regulated and 31 were down-regulated (Table 5). These biomarkers reflected differences in metabolites in mice with cognitive impairment. Compared with these 36 metabolites, 16 potential biomarkers were identified in the peppermint essential oil group, in which 14 were up-regulated and 2 were down-regulated.

### 3.4. Metabolic Pathway Analysis

**Figure 4:**
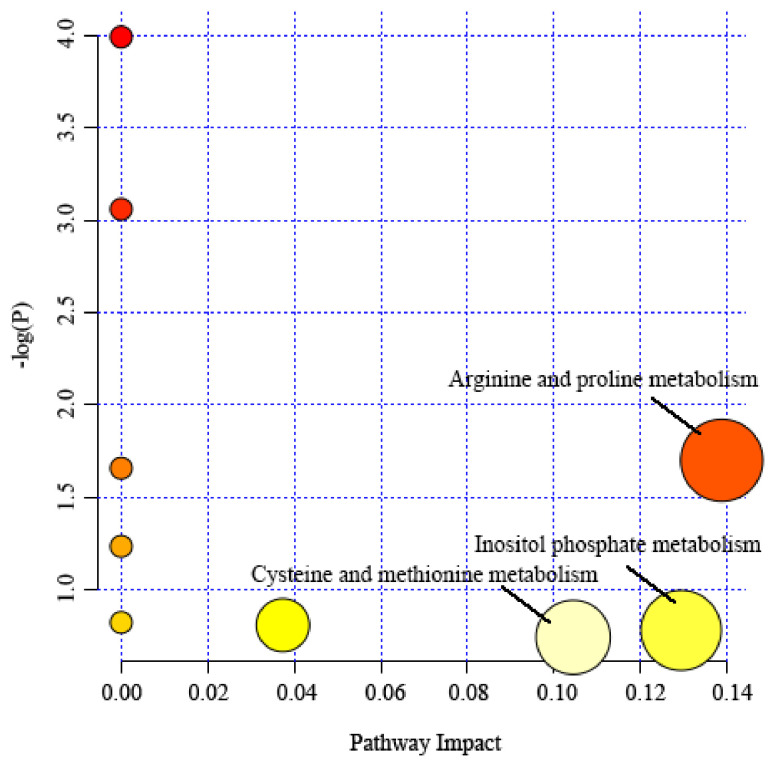
Metabolic pathway analysis of serum samples. Each circle in the figure represents a metabolic pathway. The color and size of the circle represent the *p* value and influence value of different metabolic pathways. The more red the color is, the more significant the result is, and the larger the circle is, the greater the influence value is.

The differential metabolites screened from serum samples were respectively input into the MetaboAnalyst 5.0 platform to determine the important metabolic pathways related to learning and memory impairment. Results show that differential metabolites mainly affected (Pathway Impact > 1) the metabolic pathways of arginine and proline metabolism, inositol phosphate metabolism, and cysteine and methionine metabolism (Figure 4).

### 3.5. Composition of Peppermint Essential Oil

Under the experimental conditions, the main components of peppermint essential oil used in the experiment were menthol (45.56%) and menthone (20.9%). The two main components accounted for 66.46% of the total content of essential oil. The secondary ingredients were: menthol acetate (6.64%), 1 mint 8-cineole (4.77%), new menthol (3.27%), iso-menthone (3.08%), menthofuran (2.05%), β-caryophyllene (1.79%), limonene (1.48%), pulegone (1.31%), germacrene D (1.17%), etc.; the nine secondary components listed accounted for 25.56% of the total content of essential oil. The 11 primary and secondary components accounted for 92.02% of the total essential oil content (Table 6).

## 4. Discussion

### 4.1. Behavioural Experiment

In this study, APP/PS1 transgenic mice were selected to establish an AD model. The results of the Morris water maze and shuttle box test show that peppermint essential oil sniffing could significantly improve the learning and memory ability and cognitive impairment of AD model mice.

### 4.2. Observation and Detection of Pathological Characteristics of Brain Tissue in Mice

Aβ is closely related to the pathogenesis of AD. The senile plaque formed by abnormal aggregation of Aβ has neurotoxicity. Aβ precipitation is often distributed in the hippocampus and cerebral cortex equal to learning and memory-related brain areas, thus impairing patients’ learning, memory, and cognitive ability [15,16]. The pathological changes in the cerebral cortex and hippocampus in the right hemisphere of APP/PS1 transgenic mice were observed by immunohistochemical experiment in this study. The results show that Aβ precipitation decreased and the number and morphology of neurons returned to the normal level after sniffing peppermint essential oil. Oxidative stress is a typical pathological feature of brain tissue in patients with AD. The brain is mostly composed of lipids and has a high rate of oxygen consumption, so it is vulnerable to reactive oxygen species (ROS) [17]. Malondialdehyde (MDA) is the final product of lipid peroxidation in vivo, and its content can indirectly reflect the degree of ROS attack on cells [18]. Superoxide dismutase (SOD) and glutathione peroxidase (GSH-PX) can eliminate ROS in the body, and its activity can reflect the antioxidant capacity of the body [19,20]. The results of this study show that the content of MDA in the brain tissue of APP/PS1 transgenic mice increased significantly, while the activities of SOD and GSH-PX decreased significantly, which indicate that there was peroxidation damage in the brain tissue of APP/PS1 transgenic mice. After sniffing peppermint essential oil, the content of MDA in the brain tissue of mice decreased significantly, while the activities of SOD and GSH-PX increased. The results above show that sniffing peppermint essential oil can treat AD to a certain extent by reducing Aβ precipitation in brain, protecting neuronal cells and returning them to the normal state, while alleviating peroxidation damage in brain tissue.

### 4.3. Serum Metabonomics of APP/PS1 Transgenic Mice

In this study, GC-MS combined with principal component analysis was used to study the serum metabonomics of AD model mice treated with peppermint essential oil. Results show that compared with the normal group, the serum proline, leucine, isoleucine, and methionine in the model group were significantly down-regulated, which suggests that the amino acid metabolism and energy metabolism of AD model mice were out of order. The sniffing of peppermint essential oil mainly affected three metabolic pathways: arginine and proline metabolism, inositol phosphate metabolism, and cysteine and methionine metabolism.

#### 4.3.1. Amino Acid Metabolism

Amino acids are the basic units of protein, which participate in the synthesis of many life activities and play important roles in nerve conduction, learning, memory, and receptor function [21]. The disorder of amino acid metabolism can have a potential impact on the central nervous system and can lead to neuronal damage in the brain region, resulting in cognitive disorders such as memory and learning [22]. After sniffing peppermint essential oil, the above amino acid metabolites were up-regulated and close to the normal level. Among them, leucine has been reported to improve the cognitive function of the frail elderly when combined with vitamin D [23].

#### 4.3.2. Arginine and Proline Metabolism

Arginine is an essential amino acid in the human body, which can synthesize NO through nitric oxide synthase (NOS) [24]. As an intercellular messenger and neurotransmitter, nitric oxide plays an important role in the cardiovascular system, central nervous system, and peripheral transmission [25]. Proline is not only an ideal osmotic regulator but also a protective substance for membranes and enzymes and a free radical scavenger. Arginine and proline are closely related to cardiovascular structure and function, and their levels in plasma often change due to intimal lesions [26]. Peppermint essential oil sniffing can regulate the metabolic pathways of arginine and proline and reduce the stress injury of blood vessels. It helps to reshape the injured site of blood vessels.

#### 4.3.3. Cysteine and Methionine Metabolism

Methionine is an important amino acid involved in protein synthesis. Methionine deficiency will hinder protein synthesis in the body and cause damage to the body. At the same time, methionine protects against membrane lipid peroxidation caused by oxygen free radicals in many ways, and membrane lipid peroxidation causes damage to important organelles such as cell and mitochondrial membranes [27]. Methionine and choline have also been reported to reverse cognitive and NR1 defects caused by Pb2+ exposure [28].

#### 4.3.4. Inositol Phosphate Metabolism

The results show that the content of myo-inositol in the serum of mice increased to the normal level after sniffing peppermint essential oil. Myo-inositol is mainly found in muscles, heart, lungs, and liver. It has the effect of lowering cholesterol and sedation. Mounting evidence indicates that excessive cholesterol accumulates in Alzheimer’s disease (AD), where it may drive AD-associated pathological changes [29]. Myo-inositol also plays an important role in the supply of brain cell nutrition [30].

### 4.4. Composition, Efficacy, and Safety of Peppermint Essential Oil

The highest content of peppermint essential oil in this study is menthol, accounting for 45.56% of the total content. Menthol is extracted from the stems or leaves of mint, which is the main component of most varieties of mint essential oil. Menthol can make people feel cold because it can activate the sensory nerve cold receptor TRPM8. Thus, it is used in seasoning or aromatic agents and in many drugs, daily necessities, beverages, etc. [31]. Xiao-Bing Zhang et al. found that menthol acts on hippocampal neurons and selectively enhances the affinity of hippocampal neurons in rats [31]. The side effects of peppermint essential oil are claimed to be usually mild and minimally toxic. Hepatorenal toxicity may occur only in a high dose of oral administration [32]; it is safe when used at a concentration of <3% by sniffing.

### 4.5. Prospect

At present, there are few studies on the effect of essential oils on learning and memory, and the research on its mechanism is even rarer, as most studies stay on the discovery of the curative effect. Previous studies on the mechanism of the effects of peppermint on learning and memory are mostly focused on the neurotransmitters or the effects of single compounds such as menthol or menthone [13,33]. It is speculated that menthol can improve the learning and memory ability of mice, but whether it is the role of this single component or the combined effect of multiple components remains to be confirmed by further experimental studies. This study explored the mechanism of the effect of peppermint essential oil, a natural product, on learning and memory from the perspectives of oxidative stress and metabolism, which is a new attempt. In this research, differential metabolites and metabolic pathways were found. The specific changes in differential metabolites in detail can be further studied, and the differential metabolites can be further analysed by targeted metabolomics.

## 5. Conclusions

In summary, sniffing peppermint essential oil can improve the learning and memory ability of APP/PS1 transgenic mice. It can protect brain nerves by improving amino acid metabolism and energy metabolism, reducing brain oxidative damage, and protecting neurons. Through the results of metabonomic studies, it is speculated that it may improve AD by regulating arginine and proline metabolism, inositol phosphate metabolism, and cysteine and methionine metabolism. It is speculated that menthol in peppermint essential oil plays a key role. Therefore, we will continue to conduct more in-depth research on the neurobiological mechanism of peppermint essential oil in the treatment of AD, so as to provide new ideas for the development of safe and effective new drugs for the treatment of AD.

## Figures and Tables

**Table 1:** Results of positioning cruise test ($\bar{x}$ ± S, *n* = 8).

Groups	Escape Latency/s				
	D1	D2	D3	D4	D5
Normal	94.92 ± 9.41	75.18 ± 7.86	79.48 ± 3.23	55.61 ± 9.27	43.33 ± 3.39
Model	114.27 ± 2.70	104.82 ± 9.51	110.24 ± 4.27 *	95.68 ± 5.02 *	79.14 ± 5.88 *
Positive	120.00 ± 0.00	82.12 ± 6.02	85.47 ± 3.65 ^#^	60.32 ± 8.02 ^#^	55.12 ± 2.56
Peppermint	87.99 ± 6.19	84.50 ± 4.02	85.24 ± 5.08 ^#^	64.10 ± 6.57 ^#^	57.70 ± 0.65

Note: * *p* < 0.05, compared with the normal group. ^#^ *p* < 0.05, compared with the model group.

**Table 2:** Results of space exploration test ($\bar{x}$ ± S, *n* = 8).

Groups	Residence Time in Original Platform Quadrant/s	Number of Times Crossing the Original Platform
Normal	32.87 ± 8.70	4.50 ± 0.45
Model	24.64 ± 6.58 *	1.13 ± 0.13 *
Positive	31.97 ± 5.13 ^#^	3.13 ± 0.36 ^#^
Peppermint	31.28 ± 2.45 ^#^	3.38 ± 0.60 ^#^

Note: * *p* < 0.05, compared with the normal group. ^#^ *p* < 0.05, compared with the model group.

**Table 3:** Results of shuttle box test ($\bar{x}$ ± S, *n* = 8).

Groups	Number of Active Escapes	Latency of Active Escape/s
Normal	6.00 ± 1.93	2.62 ± 0.42
Model	2.75 ± 0.28 *	3.40 ± 0.31 *
Positive	5.13 ± 1.46 ^#^	2.77 ± 0.67 ^#^
Peppermint	4.75 ± 1.75 ^#^	2.80 ± 0.44 ^#^

Note: * *p* < 0.05, compared with the normal group. ^#^ *p* < 0.05, compared with the model group.

**Table 4:** Levels of oxidative stress factors in brain tissues of each group of mice ($\bar{x}$ ± S, n = 8).

Groups	MDA (nmol/mgprot)	SOD (U/mgprot)	GSH-PX (U/mgprot)
Normal	5.01 ± 1.44	146.07 ± 18.85	84.05 ± 14.10
Model	17.71 ± 1.36 *	81.35 ± 10.49 *	36.91 ± 5.85 *
Positive	8.11 ± 1.60 ^#^	122.75 ± 15.98 ^#^	70.32 ± 6.69 ^#^
Peppermint	8.21 ± 2.20 ^#^	119.62 ± 12.78 ^#^	68.00 ± 6.84 ^#^

Note: * *p* < 0.05, compared with the normal group. ^#^ *p* < 0.05, compared with the model group.

**Table 5:** Changes in the levels of metabolites in serum samples between normal, model, peppermint essential oil group, and positive group (VIP > 1 and *p* < 0.05).

Differential Metabolites	Normal Group vs. Model Group			Model Group vs. Positive Group			Model Group vs. Peppermint Essential Oil Group		
	VIP	*p*-value	Trend	VIP	*p*-Value	Trend	VIP	*p*-Value	Trend
Glycine	3.64	9.68 × 10^−6^	↓	2.61	1.52 × 10^−3^	↓	2.81	2.75 × 10^−6^	↓
Palmitic acid	1.98	3.28 × 10^−2^	↓	3.05	4.45 × 10^−3^	↓			
Stearic acid	1.23	9.60 × 10^−3^	↓	1.11	1.60 × 10^−2^	↓			
L-Allothreonine	1.06	5.94 × 10^−3^	↓						
6-(2-Aminopropyl)benzofuran	1.11	1.29 × 10^−3^	↓						
2-hydroxybutanoic acid	1.16	1.51 × 10^−2^	↓						
Threonine	1.08	2.29 × 10^−3^	↓						
Proline	5.24	1.06 × 10^−2^	↓				5.41	1.52 × 10^−2^	↑
2-Hydroxy-3-methylbutyric acid	1.16	4.70 × 10^−3^	↓						
Succinic acid	1.12	1.65 × 10^−5^	↓						
Alanine	1.16	2.33 × 10^−3^	↓						
Cyclopentene-4-carboxylic acid, 1-(trimethylsilyl)oxy-,methyl ester	1.25	1.41 × 10^−2^	↓						
Oxoproline	6.89	5.84 × 10^−4^	↓						
Leucine	5.06	8.63 × 10^−3^	↓				5.05	2.22 × 10^−2^	↑
Isoleucine	3.97	6.17 × 10^−3^	↓				3.92	2.84 × 10^−2^	↑
N-(2-Acetamido)iminodiacetic acid	1.20	5.50 × 10^−5^	↑				1.74	1.36 × 10^−3^	↑
Sedoheptulose	2.45	3.92 × 10^−2^	↓				4.69	1.80 × 10^−2^	↑
Methionine	3.41	2.74 × 10^−2^	↓				3.30	2.79 × 10^−2^	↑
Phenylalanine	1.53	1.84 × 10^−2^	↓						
Myo-inositol	1.52	6.67 × 10^−3^	↓				2.47	2.17 × 10^−4^	↑
Octanal	1.84	3.05 × 10^−2^	↓						
Octanoic acid, di (tert-butyl)silyl ester	1.10	1.49 × 10^−5^	↑				1.17	2.76 × 10^−4^	↑
trans-4-Hydroxy-L-proline	1.31	7.02 × 10^−4^	↓				1.40	9.23 × 10^−4^	↑
O-Desmethylnaproxen	2.07	5.55 × 10^−5^	↑				3.29	6.88 × 10^−7^	↑
L-Aspartic acid	1.30	4.04 × 10^−5^	↑	1.99	4.09 × 10^−5^	↑	3.03	5.30 × 10^−10^	↑
L-Proline	1.62	8.09 × 10^−3^	↓						
Phosphate	10.49	1.64 × 10^−3^	↓	11.99	4.93 × 10^−3^	↑			
Methylmalonic acid	3.17	2.11 × 10^−2^	↓	7.89	3.42 × 10^−5^	↑			
2-Imino-6-mercapto-4,4-Dimethyl-1,2,3,4-tetrahydro-pyridine-3,5-dicarbonitrile	1.26	1.98 × 10^−2^	↓						
Pentasiloxane-dodecamethyl	1.05	4.96 × 10^−3^	↓				1.91	1.94 × 10^−3^	↓
4-Aminobutanoic acid	1.35	5.45 × 10^−5^	↓						
Adonitol	2.06	3.20 × 10^−2^	↓						
Maltose Monohydrate	1.57	3.29 × 10^−2^	↓				2.03	4.02 × 10^−3^	↑
Monolaurin derivative	1.64	1.82 × 10^−6^	↑	1.70	1.87 × 10^−2^	↑	2.80	2.25 × 10^−5^	↑
2-Butene-1,4-diol	2.41	4.29 × 10^−3^	↓						
4-Acetoxy-N, N-Methylisopropyltryptamine	1.23	6.56 × 10^−3^	↓				1.27	1.70 × 10^−2^	↑

**Table 6:** Main chemical composition and the relative content in Peppermint essential oil.

No.	Retention Time (min)	Component	Relative Content (%)
1	5.556	Isopentyl alcohol	0.02
2	12.647	α-Thujene	0.03
3	13.083	α-Pinene	0.5
4	15.667	Sabinene	0.86
5	16.265	Myrcene	0.11
6	16.741	3-Octanol	0.18
7	17.396	α-Phellandrene	0.02
8	18.065	α-Terpinene	0.22
9	18.577	p-Cymene	0.11
10	18.863	Limonene	1.48
11	19.095	1,8-Cineole	4.77
12	19.237	cis-β-Ocimene	0.15
13	19.943	trans-β-Ocimene	0.04
14	20.776	γ-Terpinene	0.37
15	21.622	cis-Sabinene hydrate	0.46
16	22.612	Terpinolene	0.1
17	23.596	Linalool	0.19
18	23.749	trans-Sabinene hydrate	0.08
19	24.188	2-Methyl butyl isovalerate	0.06
20	24.919	3-Octyl acetate	0.04
21	25.383	cis-para-Menth-2-en-1-ol	0.03
22	27.081	trans-Chrysanthemal	0.07
23	27.709	Menthone	20.9
24	28.127	Menthofuran	2.05
25	28.272	Isomenthone	3.08
26	28.591	Neomenthol	3.27
27	29.289	Menthol	45.56
28	29.388	Terpinen-4-ol	0.82
29	29.929	Isomenthol	0.59
30	30.119	Neoisomenthol	0.15
31	30.369	α-Terpineol	0.24
32	33.344	Pulegone	1.31
33	33.763	Carvone	0.03
34	34.429	Piperitone	0.5
35	35.603	Neomenthyl acetate	0.28
36	36.813	Menthyl acetate	6.64
37	37.855	Isomenthyl acetate	0.2
38	42.504	α-Copaene	0.04
39	43.038	α-Bourbonene	0.33
40	43.389	β-trans-Elemene	0.07
41	45.367	β-Caryophyllene	1.79
42	46.004	β-Gurjunene	0.05
43	47.390	trans-β-Farnesene	0.22
44	47.641	α-Humulene	0.08
45	49.235	Germacrene D	1.17
46	50.124	Bicyclogermacrene	0.2
47	50.302	γ-Amorphene	0.04
48	51.475	delta-Cadinene	0.07
49	56.093	Viridiflorol	0.11

## Sample Availability

Samples of the compounds are available from the authors.
